# Reduced Retinal Perfusion With Preserved Microvascular Network Density in Early Alzheimer's Disease: A Multimodal Imaging Study

**DOI:** 10.1167/tvst.15.8.7

**Published:** 2026-08-11

**Authors:** Giovana Rosa Gameiro, Andrew Hoover, Paulo Schor, Hong Jiang, Jianhua Wang

**Affiliations:** 1 Department of Ophthalmology, Bascom Palmer Eye Institute, University of Miami Miller School of Medicine, Miami, FL, USA; 2 Department of Ophthalmology and Visual Sciences, Federal University of São Paulo–EPM/UNIFESP, São Paulo, SP, Brazil; 3 Duke University School of Medicine, Durham, NC, USA; 4 Department of Neurology, University of Miami Miller School of Medicine, Miami, FL, USA

**Keywords:** alzheimer's disease, retinal blood flow, microvasculature, perfusion, mild cognitive impairment, optical coherence tomography, retina, biomarkers

## Abstract

**Purpose:**

To characterize retinal neurovascular alterations in Alzheimer's disease (AD) and mild cognitive impairment (MCI) and examine structure–function relationships.

**Methods:**

Eighty-two participants (28 with AD, 21 with MCI, and 33 cognitively normal controls) underwent retinal imaging. Retinal blood flow (RBF) was measured using a function imager. Retinal vessel density (RVD), retinal vessel length density (RVLD), vessel width, and capillary perfusion density (CPD) were quantified with optical coherence tomography (OCT) angiography and retinal tissue volume (RTV) with ultra–high-resolution OCT. Derived metrics included retinal tissue perfusion (RTP), retinal capillary flow index (RCF), and volumetric vessel density. Group comparisons and age-adjusted correlations were performed.

**Results:**

Compared with controls, the combined AD + MCI group had significantly lower RBF (3.18 ± 1.02 vs. 4.22 ± 0.79 nL/s; *P* < 0.001), RCF (0.21 ± 0.07 vs. 0.26 ± 0.04 nL/s/mm; *P* < 0.001), and RTP (2.89 ± 0.94 vs. 3.86 ± 0.77 nL/s/mm^3^; *P* < 0.001). Morphometric indices of the perfused capillary network and retinal tissue volume (RVD, RVLD, CPD, vessel width, RTV) did not differ (all *P* > 0.05). In AD + MCI, RBF was not associated with structural or morphometric microvascular metrics, whereas controls exhibited age-adjusted correlations between RBF and vessel density and length density.

**Conclusions:**

AD and MCI are characterized by reduced retinal perfusion and capillary flow index despite preserved network density and retinal tissue volume, suggesting altered retinal hemodynamic–morphometric relationships.

**Translational Relevance:**

Hemodynamic retinal imaging detects early perfusion impairment in AD and MCI before changes in microvascular network density or retinal tissue volume, offering a noninvasive biomarker for detection and therapeutic monitoring.

## Introduction

Alzheimer's disease (AD) is a complex and multifactorial neurodegenerative disorder with a gradual onset and slow progression.^1^^,^^2^ It is marked by an irreversible decline in cognitive and functional abilities, with limited treatment options currently available.^3^ Mild cognitive impairment (MCI) is an intermediate stage between normal cognitive function and AD.^4^ It is characterized by a clinically evident decline in one or more cognitive areas that exceeds what is typical for the individual's age, with minimal or no difficulty in performing instrumental activities of daily living.^5^ Globally, more than 55 million individuals are affected by AD and related dementias, and this number is expected to rise to 139 million by 2050, resulting in significant emotional, physical, and financial burdens.^3^

Previous studies indicate that vascular changes, particularly at the capillary level, play a critical role in the pathogenesis of AD,^6^^,^^7^ and there is ongoing debate about whether reduced blood flow is a contributor or a consequence of the disorder.^2^ These vascular impairments are crucial for understanding the disease's progression, as decreased cerebral perfusion is observed not only during the clinical stages of AD but also in the preclinical phase, before the onset of cognitive decline.^2^^,^^8^ Indeed, cerebral vascular dysfunction is a potential biomarker for identifying the disease in its preclinical stages.^2^

While magnetic resonance imaging (MRI) and transcranial Doppler imaging can examine the brain's microvasculature, they are costly and time-consuming and, in some cases, require contrast agents or radiotracers, thereby limiting their feasibility for repeated or large-scale assessments.^4^^,^^6^ In addition, direct in vivo visualization of cerebral capillary networks remains challenging.^9^ A viable alternative is to image the retinal microvasculature, which is more accessible and cost-effective.^3^ The retina is often considered a surrogate of central nervous system tissue, given its shared embryologic origin and close anatomic and physiologic parallels with the brain,^4^ offering an opportunity to monitor and understand the intricate structural and molecular processes in the patient's brain.^3^

The retina has been previously utilized to investigate vascular changes^6^^,^^7^^,^^10^ and neurodegeneration in patients with AD.^4^ Studies have demonstrated decreased retinal blood flow rate,^10^ decreased retinal tissue perfusion,^7^ and focal thinning of the ganglion cell–inner plexiform layer (GCIPL).^4^

Despite these promising results, the literature on retinal vessel density in AD and MCI remains inconsistent. Several optical coherence tomography angiography (OCTA) studies report reduced macular or peripapillary vessel density in patients with cognitive impairment, suggesting retinal capillary rarefaction.^11^^–^^13^ However, other investigations, including more recent biomarker-characterized or methodologically rigorous cohorts, have found minimal or no differences in vessel density between AD, MCI, and cognitively normal individuals.^14^^,^^15^ These discrepancies likely stem from substantial heterogeneity in diagnostic criteria, disease severity, systemic vascular comorbidities, OCTA scan size, segmentation boundaries, and image-processing algorithms. Consequently, it remains uncertain whether static retinal vessel density reliably reflects AD-related microvascular pathology or whether early vascular involvement may instead present as hemodynamic dysregulation before the onset of structural capillary loss.

Furthermore, understanding the complex relationships among retinal blood flow (RBF), retinal vessel density (RVD), retinal vessel length density (RVLD), capillary perfusion density (CPD), and retinal tissue volume (RTV), as well as novel derived metrics such as volumetric vessel density (VVD), retinal capillary flow index (RCF), and retinal tissue perfusion (RTP), is essential for unraveling the multifaceted neurovascular changes in AD and MCI. Critically, bulk macular blood flow (RBF), measured by the retinal function imager (RFI), and capillary network density metrics (RVD, RVLD), derived from OCTA, capture distinct vascular compartments and have been shown to be statistically independent of one another, highlighting the necessity of measuring each parameter separately.^16^

Investigating these parameters together and comparing them to cognitively normal controls provides a more comprehensive view of retinal neurovascular health and its potential as a window into cerebral pathology. This integrative approach advances the field of oculomics, a field dedicated to developing rapid, noninvasive, and cost-effective biomarkers for screening, diagnosing, and stratifying risks of systemic diseases, ultimately guiding treatment prioritization.^17^

By exploring the distinct and overlapping patterns of these retinal biomarkers, this study aims to deepen our understanding of retinal hemodynamic and morphometric alterations across disease stages and provide insight into neurovascular changes in AD and MCI, supporting the future development of retinal imaging biomarkers.

## Methods

A total of 82 participants were recruited for this study, including 28 individuals diagnosed with AD, 21 with MCI, and 33 cognitively healthy controls (NCs). This study was approved by the institutional review board for human research at the University of Miami (UM #20070492), and written informed consent was obtained from each participant. All patients were treated in accordance with the principles of the Declaration of Helsinki. One eye from each participant was scanned. The right eye was prioritized for imaging; the left eye was selected if the right eye did not meet the eligibility criteria.

Three imaging modalities were used to assess RBF, vessel density (VD), vessel length density (VLD), and RTV across the AD, MCI, AD + MCI combined, and control groups. Additional derived parameters, including VVD, RCF, and RTP, were also analyzed.

Patients with AD and MCI were recruited at the Division of Cognitive Disorders within the Department of Neurology at the University of Miami, a tertiary referral clinic specializing in cognitive disorders. Diagnoses of AD and MCI were based on the National Institute on Aging–Alzheimer's Association criteria and confirmed through a consensus conference involving neurologists, psychiatrists, and neuropsychologists. During routine clinical visits, eligible patients were referred to our retinal imaging laboratory for participation in the study. As part of their clinical evaluation, all patients had undergone brain MRI to exclude major cerebrovascular disorders. Control participants were recruited from the Bascom Palmer Eye Institute and consisted of staff members or family members of patients who volunteered to participate in the study.

All participants underwent slit-lamp biomicroscopy prior to imaging to ensure clear optical media. Only scans meeting predefined quality criteria (OCTA signal strength ≥7) were included in the analysis, ensuring that media opacity did not affect image acquisition or quantitative measurements. Ophthalmic exclusion criteria were as follows: refractive errors exceeding +6.0 or −6.0 diopters, age-related macular degeneration, diabetic retinopathy, glaucoma, cystic macular edema, cataracts, and corneal diseases. Additionally, individuals with a history of stroke, coagulopathy, uncontrolled hypertension, or uncontrolled diabetes were also excluded. Images were also reviewed for retinal abnormalities, and any case showing structural or vascular pathology on optical coherence tomography (OCT), OCTA, or RFI was excluded from analysis.

### RBF Assessment With RFI

**Figure 1.**
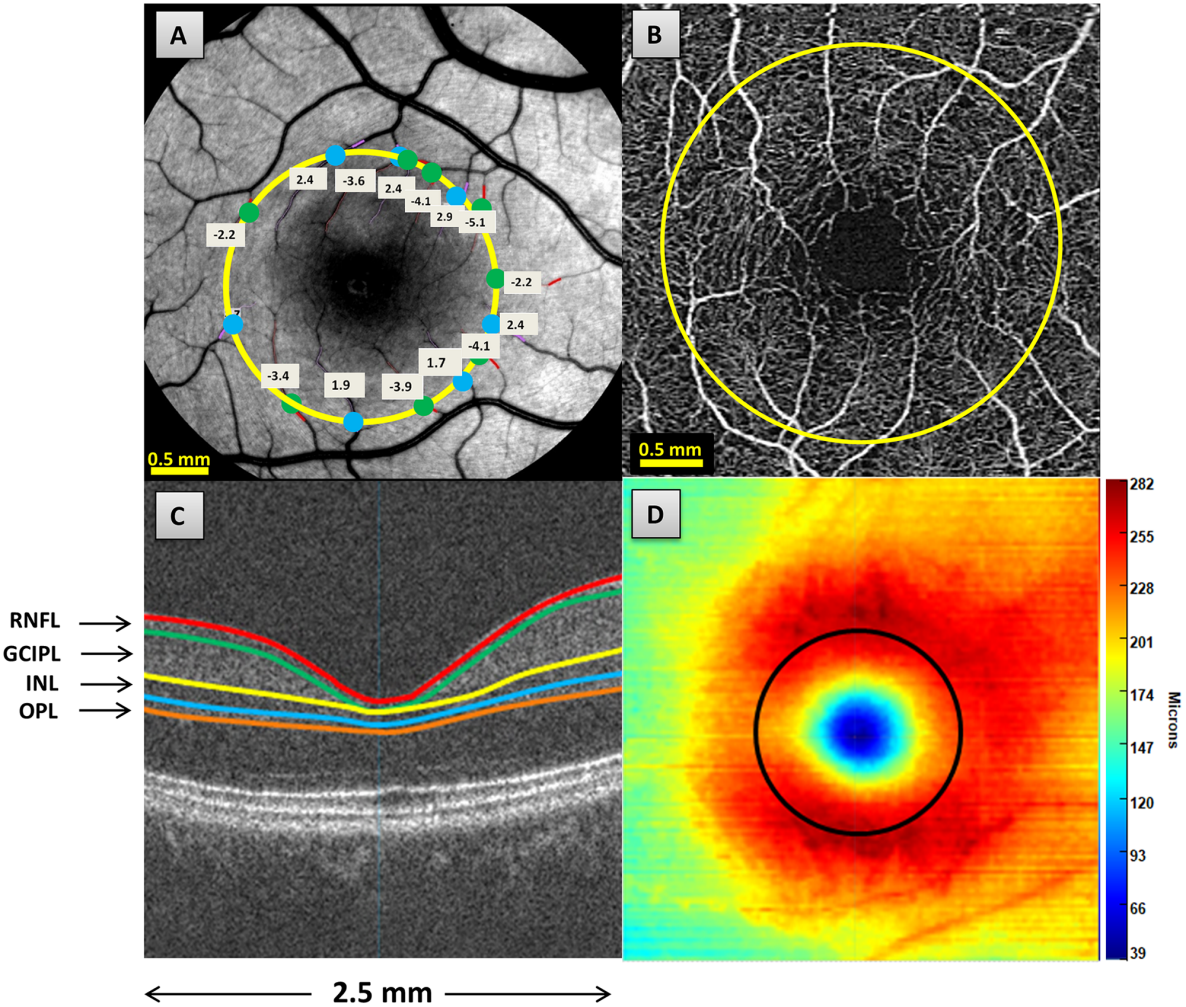
(**A**) Retinal blood flow velocity (BFV) was captured using RFI, focusing on a 20° field of view. Arterioles are highlighted in *red* and their corresponding blood flow velocities (in mm/s) overlaid. Venules and their velocities are indicated in *pink*. A negative value signifies blood flow moving away from the heart, observed in the arteriolar flow directed toward the fovea, while a positive value represents flow toward the heart, associated with venules. Vessel diameters were measured where they intersected a circle with a radius of 2.5 mm centered on the fovea, and these measurements were used to determine blood flow in arterioles (*green dots*) and venules (*blue dots*). The total macular blood flow volume for arterioles was calculated by summing the volumes of each arteriole crossing the circle, while the same method was applied to venules. (**B**) A 3-mm × 3-mm angiographic image of the total retinal vascular network (RVN) with the analyzed area of a 2.5-mm disc (*yellow circle*). (**C**) The intraretinal layers were visualized using UHR-OCT and segmented with Orion software. (**D**) The thickness map of the inner retina, including the RNFL, GCIPL, INL, and OPL, was measured within a disc of 2.5 mm in diameter.

RBF was measured using the RFI (Optical Imaging, Rehovot, Israel; Fig. 1). The details of the retinal function imaging system and its applications have been extensively reviewed in prior studies.^7^^,^^10^^,^^18^ The RFI system uses a noninvasive fundus camera equipped with stroboscopic illumination, utilizing hemoglobin as a contrast agent. In this study, pupil dilation was performed prior to each imaging procedure. Retinal blood flow was tracked by monitoring the movement of red blood cells, which was then used to calculate blood flow velocity. Measurements were taken separately for arterioles and venules within a 2.5-mm circle centered on the fovea, and the blood flow measurements (calculated as nL/s) from both arterioles and venules were averaged to represent RBF.^6^^,^^7^

### RTV Assessment With Custom Ultra-High-Resolution OCT

Custom ultra-high-resolution OCT (UHR-OCT; Fig. 1) was used to measure the tissue volume of intraretinal layers, with the system configuration detailed in previous studies.^7^^,^^18^ This spectral-domain OCT system features an axial resolution of approximately 3 µm and a scanning speed of 24,000 A-scans per second. It employs a raster scan technique that covers a 6-mm × 6-mm field centered on the fovea, capturing volumetric data through a protocol consisting of 128 consecutive B-scans, each containing 512 A-scans.^7^

The resulting dataset was analyzed using specialized software (Orion, Voxeleron LLC, Pleasanton, CA, USA.) to segment and quantify the volume of each of the six intraretinal layers: retinal nerve fiber layer (RNFL), GCIPL, inner nuclear layer (INL), outer plexiform layer (OPL), outer nuclear layer, and retinal photoreceptors (PRs). In addition to these individual layer volumes, total retinal thickness (TRT), encompassing all retinal layers from the inner limiting membrane to the PR layer, was also measured. Given that the retinal vasculature extends across the inner retina from the RNFL to the OPL,^7^ the tissue volume encompassing these four layers (RNFL, GCIPL, INL, and OPL) was also quantified. The RTV within a 2.5-mm diameter area centered on the fovea was analyzed to align with blood flow measurements taken in the same 2.5-mm circular area using the RFI, as well as with RVD and RVLD measurements obtained using OCTA.

### RVD and RVLD Assessment Using OCTA

RVD and RVLD were quantified from OCTA scans acquired using a commercial device (Carl Zeiss Meditec, Dublin, CA, USA; Fig. 1) with a 3-mm × 3-mm macular scan centered on the fovea. Only images with a quality score ≥7 (on a 10-point scale) were included to ensure image reliability.

OCTA images were resampled to 1024 × 1024 pixels and processed using a custom MATLAB-based software pipeline (MathWorks, Natick, MA, USA), as previously described.^16^ Briefly, grayscale inversion and correction for nonuniform illumination were applied, followed by morphologic opening to remove background noise and nonvascular structures, yielding a binary vessel map. Vessels with a diameter ≥25 µm were classified as large vessels and excluded from analysis. The remaining small vessels were retained and skeletonized to generate a vessel-length map. OCTA detects signal from moving red blood cells within a velocity-dependent detection window^19^; accordingly, RVD and RVLD reflect the density and spatial organization of the perfused small vessel network (<25 µm) and are interpreted as morphometric indices of the capillary bed rather than as direct measures of volumetric flow.^16^

**Figure 2.**
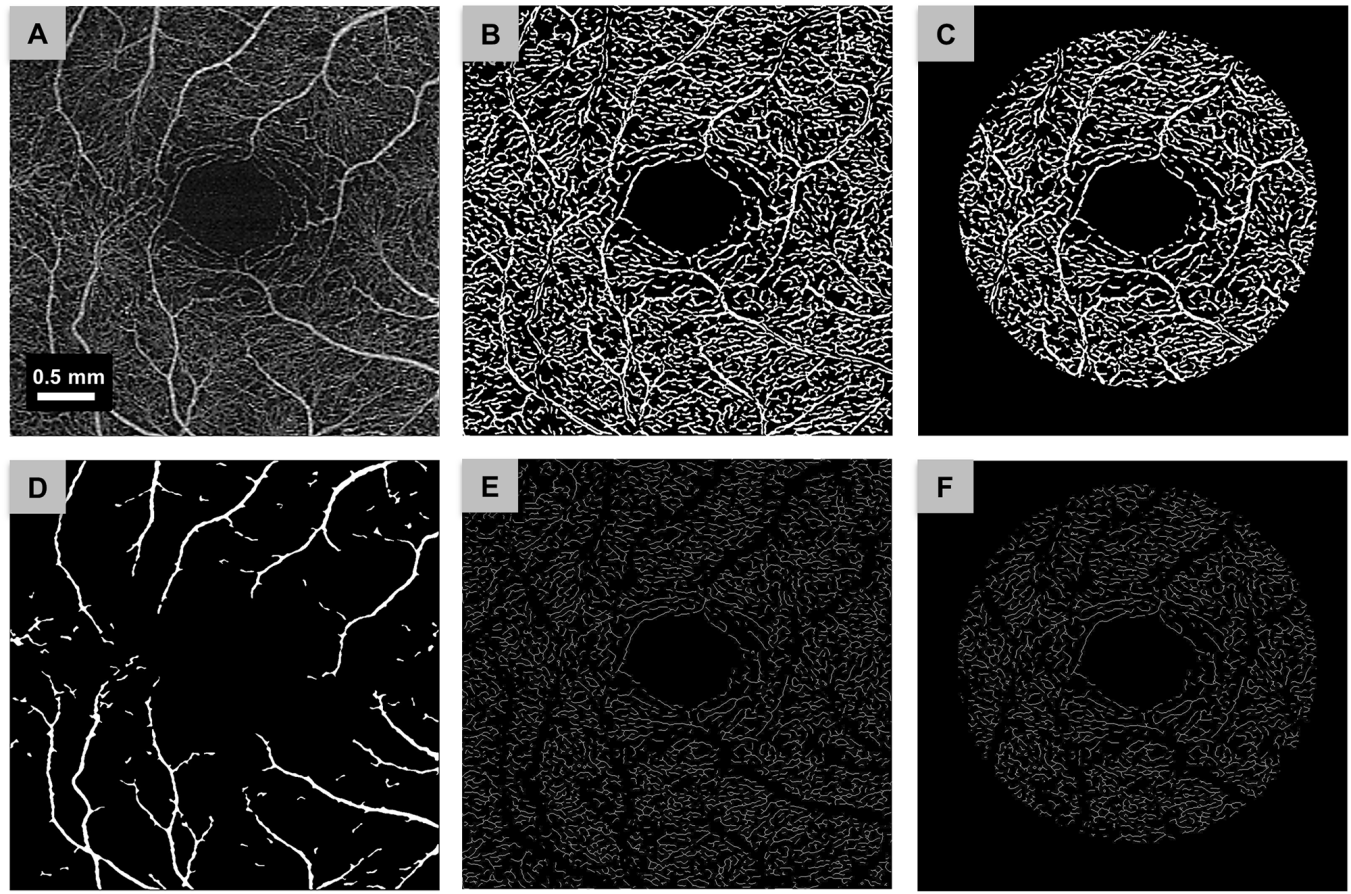
Segmentation and partitioning of the retinal vascular network. (**A**) En face projection of the retinal vasculature. (**B**) Binarized vessel map following grayscale inversion, nonuniform illumination correction, background suppression, and morphological filtering. (**C**) Definition of an annular region of interest (ROI) centered on the fovea (inner diameter: 0.6 mm; outer diameter: 2.5 mm). This was used for the RVD calculation. (**D**) Extraction of large vessels (diameter ≥25 µm). (**E**) Skeletonization of small vessels obtained by subtracting large vessels from the binarized vasculature map. (**F**) Final annular ROI containing only small vessels (diameter <25 µm), used for quantitative microvascular analysis of the RVLD and CPD.

The center of the fovea was automatically detected, and an annular region (inner diameter: 0.6 mm; outer diameter: 2.5 mm) was applied to both the binary and skeletonized images to isolate the parafoveal capillary network (Fig. 2). Quantification was performed using ImageJ (version 1.54g; National Institutes of Health, Bethesda, MD, USA). RVD was defined as the percentage of the annular area occupied by vessels, while RVLD was defined as the total vessel length (mm) per unit area (mm²), expressed as mm^−1^.^16^^,^^20^ Mean vessel width was then estimated as the ratio of RVD to RVLD, which reflects the average capillary caliber across the macular region.

### CPD Assessment Using OCTA

CPD was measured using the same OCTA device (Carl Zeiss Meditec). The CPD, its imaging protocol, and analysis have been documented in previous studies.^6^^,^^21^ This technique employed a 3-mm × 3-mm scanning protocol centered on the fovea to capture angiographic en face images of the retinal vascular network, which included both superficial and deep vascular plexuses. The foveal avascular zone, a region without retinal blood vessels that measures 0.6 mm in diameter, was excluded from the density measurement. Therefore, an annulus centered on the fovea, with its outer boundary defined by a diameter of 2.5 mm, was analyzed. The acquired images underwent comprehensive processing, including segmentation to differentiate microvessels from the large vessels (diameter larger than 25 µm), which were removed, and fractal analysis to assess vessel density (Fig. 2). Fractal analysis was conducted in each sector or annular zone using the box counting method with the fractal analysis software (TruSoft Benoit Pro 2.0; TruSoft International, St. Petersburg, FL, USA). This analysis yielded the fractal dimension (Dbox), which reflects the spatial complexity and topological organization of the capillary network in each area.^6^^,^^21^

### Calculation of RTP, RCF, and VVD

To further evaluate retinal microcirculation and structure–function dynamics, three derived parameters were computed using the primary variables RBF, RVLD, RVD, and RTV. RTP (nL/s/mm^3^) was calculated as RBF divided by RTV of the four inner layers (RTP = RBF / RTV), representing the efficacy of blood supply to retinal tissue.^7^ RCF (nL/s/mm) was defined as RBF divided by RVLD (RCF = RBF / RVLD).^16^

Because RBF reflects bulk volumetric flow through arterioles and venules (≥25 µm) and RVLD reflects the length density of the perfused small capillary network (<25 µm), RCF is a cross-compartment ratio that quantifies macro-hemodynamic supply relative to the extent of the downstream microvascular bed; a reduction in RCF with preserved RVLD indicates that upstream flow is disproportionately reduced relative to a preserved capillary network. VVD (%/mm^3^) was derived by dividing RVD by RTV of the four inner layers (VVD = RVD / RTV). This metric quantifies capillary concentration relative to intraretinal tissue volume, providing a volumetric assessment of microvascular density.^22^

### Statistical Analysis

Statistical analyses were performed using IBM SPSS Statistics for Windows (version 31.0; IBM Corp., Armonk, NY, USA). Normality of continuous variables was assessed using the Shapiro–Wilk test. Group differences among AD, MCI, and NC participants were evaluated using one-way analysis of variance (ANOVA) for normally distributed variables, with Bonferroni-adjusted post hoc comparisons. For variables that deviated from normality (RVD and CPD), nonparametric tests were applied as appropriate.

For comparisons between two groups, independent samples *t*-tests or Mann–Whitney *U* tests were used depending on data distribution. The primary outcomes of interest were the hemodynamic parameters RBF, RCF, and RTP. Morphometric indices of the perfused capillary network (RVD, RVLD, CPD) and tissue volume measures (RTV, TRT) were evaluated as secondary outcomes to provide a biological context for the functional findings. Categorical variables were compared using the χ^2^ test. Correlations between retinal hemodynamic and morphometric measures were assessed using Spearman's rank correlation coefficient (ρ). Partial correlations controlling for age were performed to account for potential confounding effects of aging.

Because age differed between groups, analysis of covariance (ANCOVA) was conducted to assess group effects on RBF, RCF, and RTP while adjusting for age. A two-sided *P* < 0.05 was considered statistically significant. Effect sizes for pairwise group comparisons (AD vs. NC, MCI vs. NC, AD + MCI vs. NC, and AD vs. MCI) were calculated using Cohen's *d* based on pooled standard deviations. Effect sizes were interpreted according to conventional thresholds (small = 0.2, moderate = 0.5, large = 0.8) to quantify the magnitude of group differences.

Post hoc power analyses were performed in G*Power (version 3.1.9.7) using effect sizes derived from the omnibus group tests (ANOVA) for each outcome. Achieved power was calculated for the primary hemodynamic measures (RBF, RCF, RTP) and for vascular density–based morphometric measures (VD, VLD, VVD) to evaluate the ability of the current sample size to detect observed effects.

## Results

The baseline characteristics of the study participants are summarized in Table 1. A χ^2^ test of independence was conducted to examine the association between study group (AD, MCI, NC) and sex. The result was not statistically significant (*P* = 0.86), indicating no significant difference in sex distribution across the groups. However, a one-way ANOVA revealed a significant difference in age across the groups (*η*² = 0.182; 95% confidence interval, 0.044–0.316). Post hoc Bonferroni comparisons showed that participants in the AD group were significantly older than those in both the MCI (*P* = 0.045) and NC (*P* < 0.001) groups. No significant age difference was found between the MCI and NC groups (*P* = 0.63). Disease duration was compared only between the AD and MCI groups and did not differ significantly (independent-samples *t*-test, *P* = 0.11). Best-corrected visual acuity (BCVA) was available for the AD and MCI groups only and did not differ significantly between them (Mann–Whitney U test, *P* = 0.34).

**Table 1.** Demographics and Clinical Manifestations of AD, MCI, and Control Study Participants

Characteristic	AD	MCI	Control	*P* Value
*N*	28	21	33	—
Age, mean ± SD, y	71.4 ± 8.2	68.4 ± 6.8	62.70 ± 9.12	<0.001
Sex, male/female, *n*	13/15	9/12	13/20	0.86
Disease duration^*^, mean ± SD, y	3.7 ± 1.5	3.1 ± 1.3	—	0.11
BCVA (logMAR)^**^, mean ± SD	0.09 ± 0.13	0.04 ± 0.06	—	0.34
Smoking, *n*	0	0	0	—
Hypertension, *n*	16	10	16	0.74
Dyslipidemia, *n*	11	11	9	0.18
Diabetes, *n*	3	4	5	0.71
Coronary artery disease, *n*	5	4	1	0.11

*P* values were calculated using one-way ANOVA for continuous variables and χ^2^ tests for categorical variables. logMAR, logarithm of the minimum angle of resolution.

* Disease duration was compared between AD and MCI using an independent samples *t*-test.

** BCVA was available only for the AD and MCI groups and was compared using the Mann–Whitney *U* test.

Hemodynamic parameters differed significantly across diagnostic groups (Tables 2, 3). One-way ANOVA revealed significant group effects for RBF (*F*(2, 78) = 15.70; *P* < 0.001), RCF (*F*(2, 74) = 11.24; *P* < 0.001), and RTP (*F*(2, 75) = 14.16; *P* < 0.001). Bonferroni post hoc analyses indicated that RBF was significantly reduced in the AD group compared with NC (*P* < 0.001; Table 3) and approached significance compared with MCI (*P* = 0.05). RCF was significantly lower in AD participants compared to both NC (*P* < 0.001) and MCI (*P* = 0.01), whereas RTP was significantly reduced in AD relative to NC (*P* < 0.001) and showed a trend toward reduction compared to MCI (*P* = 0.09). Additionally, RBF was lower in MCI compared to NC (*P* = 0.03), and RTP was also reduced in MCI versus NC (*P* = 0.04).

**Table 2.** Groupwise Comparisons of Retinal Vascular, Structural, and Hemodynamic Metrics (Mean ± SD) in AD, MCI, and NC Participants

Characteristic	AD	MCI	AD + MCI	NC
RBF (nL/s)	2.90 ± 0.99	3.54 ± 0.96	3.18 ± 1.02	4.22 ± 0.79
RCF (nL/s/mm)	0.18 ± 0.06	0.24 ± 0.08	0.21 ± 0.07	0.26 ± 0.04
RTP (nL/s/mm^3^)	2.66 ± 0.88	3.22 ± 0.94	2.89 ± 0.94	3.86 ± 0.77
RVD (%)	27.49 ± 3.46	27.11 ± 4.18	27.32 ± 3.76	28.57 ± 2.32
RVLD (mm^−1^)	15.86 ± 1.86	15.49 ± 2.60	15.69 ± 2.20	16.28 ± 1.41
CPD (Dbox)	1.756 ± 0.023	1.750 ± 0.035	1.754 ± 0.028	1.760 ± 0.016
Vessel width (µm)	17.3 ± 0.8	17.6 ± 0.9	17.4 ± 0.9	17.6 ± 0.6
TRT (mm^3^)	1.97 ± 0.11	1.98 ± 0.10	1.97 ± 0.11	2.00 ± 0.11
RTV inner layers (mm^3^)	1.09 ± 0.07	1.11 ± 0.09	1.10 ± 0.08	1.10 ± 0.10
VVD (%/mm^3^)	25.29 ± 3.39	24.79 ± 4.07	25.08 ± 3.67	26.37 ± 3.24

**Table 3.** Pairwise Post Hoc Comparisons Between Diagnostic Groups

Metric	AD vs. MCI	AD vs. NC	MCI vs. NC	AD + MCI vs. NC
RBF (nL/s)	0.05	**<0.001**	**0.03**	**<0.001**
RCF (nL/s/mm)	**0.01**	**<0.001**	0.56	**<0.001**
RTP (nL/s/mm^3^)	0.09	**<0.001**	**0.04**	**<0.001**
RVD (%)	1.00	0.66	0.36	0.28
RVLD (mm^−1^)	1.00	1.00	0.46	0.19
CPD (Dbox)	1.00	1.00	1.00	0.98
Vessel width (µm)	1.00	0.81	1.00	0.47
TRT (mm^3^)	1.00	0.90	1.00	0.39
RTV inner layers (mm^3^)	1.00	1.00	1.00	0.91
VVD (%/mm^3^)	1.00	0.78	0.38	0.12

Bold font indicates significance.

In contrast, no significant group differences were observed for morphometric indices of the perfused capillary network or retinal tissue volume, including RVD, RVLD, CPD, TRT, or inner retinal layer volume, with all *P* values >0.05 (Table 3). Vessel width did not differ significantly between groups (*F*(2, 75) = 0.73; *P* = 0.49), and all pairwise comparisons were nonsignificant (all *P* > 0.70).

Comparisons between the combined AD + MCI group and the NC group highlighted significant differences. The AD + MCI group was significantly older, with a mean age of 70.14 ± 7.69 years compared to 62.70 ± 9.12 years in the NC group (*P* < 0.001). Retinal hemodynamic measures were consistently reduced in the AD + MCI group. Specifically, RBF was significantly lower in AD + MCI participants (3.18 ± 1.02 nL/s) than in controls (4.22 ± 0.79 nL/s; *P* < 0.001; Tables 2, 3). RCF was also reduced (0.21 ± 0.07 nL/s/mm vs. 0.26 ± 0.04 nL/s/mm; *P* < 0.001), as was RTP (2.89 ± 0.94 nL/s/mm^3^ vs. 3.86 ± 0.77 nL/s/mm^3^; *P* < 0.001).

In contrast, no significant differences were detected between groups in morphometric microvascular indices or retinal tissue volume, including RVD, RVLD, CPD, vessel width, TRT, or retinal tissue volume of the inner layers (all *P* > 0.05). ANCOVA controlling for age confirmed significant group effects on RBF (*P* < 0.001), RCF (*P* = 0.005), and RTP (*P* < 0.001), while age was not a significant covariate. Bonferroni-adjusted pairwise comparisons of estimated marginal means from the ANCOVA models are presented in Table 4. After adjustment for age, RBF and RTP remained significantly lower in the AD group compared with NC, and RCF was significantly reduced in AD compared with both MCI and NC, whereas no significant differences were observed between MCI and NC.

**Table 4.** Age-Adjusted Pairwise Comparisons of Retinal Hemodynamic Parameters (ANCOVA With Bonferroni Correction)

Metric	Comparison	Mean Difference	*P* Value	95% CI
RBF	AD vs. MCI	−0.58	0.09	−1.23 to 0.06
	AD vs. NC	**−1.17**	**<0.001**	−1.80 to −0.55
	MCI vs. NC	−0.59	0.08	−1.23 to 0.05
RCF	AD vs. MCI	**−0.05**	**0.02**	−0.10 to −0.01
	AD vs. NC	**−0.08**	**<0.001**	−0.12 to −0.03
	MCI vs. NC	−0.02	0.70	−0.07 to 0.02
RTP	AD vs. MCI	−0.5	0.15	−1.11 to 0.11
	AD vs. NC	**−1.02**	**<0.001**	−1.63 to −0.42
	MCI vs. NC	−0.52	0.13	−1.15 to 0.10

Bold font indicates statistically significant comparisons (P < 0.05).

Effect size analysis demonstrated large effects for hemodynamic metrics (RBF, RCF, RTP) when comparing AD and AD + MCI groups with controls, whereas morphometric microvascular density metrics showed small effect sizes, consistent with the absence of significant structural differences (Table 5).

**Table 5.** Effect Sizes (Cohen's *d*) for Pairwise Comparisons of Retinal Vascular, Structural, and Hemodynamic Metrics Among AD, MCI, and NC Groups

Variable	AD vs. CN	MCI vs. CN	AD + MCI vs. CN	AD vs. MCI
RBF	−1.49	−0.78	−1.11	−0.66
RVD	−0.36	−0.42	−0.38	0.1
RVLD	−0.26	−0.37	−0.31	0.16
VVD	−0.33	−0.43	−0.37	0.14
RCF	−1.41	−0.34	−0.84	−0.76
RTP	−1.44	−0.75	−1.1	−0.63

A post hoc power analysis was conducted using G*Power (version 3.1.9.7) based on effect sizes derived from the ANOVA results for the three-group comparison (AD, MCI, and NC). With α = 0.05, the achieved statistical power was 0.999 for RBF (*n* = 81), 0.992 for RCF (*n* = 77), and 0.998 for RTP (*n* = 78), indicating that the study was well powered to detect group differences in retinal hemodynamic parameters. In contrast, morphometric microvascular density metrics demonstrated smaller effect sizes, consistent with the absence of significant group differences in capillary network density metrics.

As expected, given that RCF and RTP are derived from RBF (RCF = RBF / RVLD; RTP = RBF / RTV), in the AD + MCI group, RBF showed strong positive correlations with both RCF (ρ = 0.88, *P* < 0.001) and RTP (ρ = 0.97, *P* < 0.001), which remained significant after adjustment for age (ρ = 0.90, *P* < 0.001; ρ = 0.97, *P* < 0.001). Scatterplots illustrating pairwise relationships among these variables are presented in Supplementary Figure S1. No other significant correlations were observed between morphometric microvascular density metrics (RVD, RVLD, CPD), vessel width, or retinal tissue volumes (RTV inner layers, TRT) and RBF or tissue volume measures, either before or after controlling for age. Additionally, neither age nor disease duration was significantly associated with any retinal vascular or thickness parameter.

In the control group, RBF was strongly and positively correlated with RCF and RTP (ρ = 0.86 and 0.75, respectively; both *P* < 0.001), and these associations remained robust after adjustment for age (ρ = 0.89 and 0.88; both *P* < 0.001). Age was negatively associated with multiple vascular and perfusion measures, including VD, VLD, RBF, and RTP (all *P* ≤ 0.03). After controlling for age, modest positive correlations emerged between RBF and both VD (ρ = 0.40, *P* = 0.04) and VLD (ρ = 0.39, *P* = 0.047). No other significant associations were observed, including with CPD, vessel width, or retinal tissue volumes.

## Discussion

A central unresolved question in the retinal oculomics literature is whether hemodynamic impairment precedes detectable changes in capillary network density in early AD and MCI, a distinction with direct implications for biomarker timing and clinical utility. To address this, we simultaneously assessed three complementary dimensions of retinal neurovascular status across the cognitive spectrum: bulk arteriolar and venular flow by RFI, capillary network morphometry by OCTA, and retinal tissue volume by UHR-OCT. Applying this integrated multimodal approach to the same cohort under standardized protocols allows determination of whether these parameters change together or independently as the disease advances. Given the close anatomic and physiologic parallels between the retina and the brain, these retinal markers may reflect broader cerebrovascular alterations associated with AD.^23^

A central finding of this study is the identification of a distinct retinal hemodynamic–morphometric signature in individuals with AD and MCI: significantly reduced RBF, RCF, and RTP, despite preserved morphometric indices of the perfused capillary network (RVD, RVLD, vessel width, CPD) and retinal tissue volume (RTV). This pattern of dissociation, in which functional deficits emerge in the absence of overt structural rarefaction, was consistent across the AD + MCI group. These hemodynamic impairments remained significant after adjusting for age (via ANCOVA), supporting their attribution to disease-related changes rather than normal aging.

Mechanistically, this dissociation between reduced bulk flow and preserved capillary network density may reflect altered regulation of arteriolar tone, rather than structural capillary rarefaction. RBF, measured by the RFI, reflects flow through arterioles and venules (≥25 µm), while RVD, RVLD, and CPD reflect the density and topological organization of the small capillary bed (<25 µm). The significant reduction in RBF, alongside preserved capillary density metrics, therefore suggests that upstream arteriolar flow is impaired, while the downstream capillary architecture, as captured by OCTA morphometry, remains largely unchanged.

Potential contributing mechanisms may include endothelial dysfunction, impaired pericyte-mediated vasomotor regulation, and altered neurovascular coupling signaling, all of which have been implicated in AD pathophysiology and would be expected to reduce arteriolar flow without necessarily causing capillary rarefaction.^24^^–^^26^

The absence of group differences in retinal vessel density in our cohort contrasts with earlier reports showing reduced macular or peripapillary vessel density in AD and MCI.^21^^,^^27^ However, several recent and methodologically rigorous studies have likewise reported no measurable reduction in vessel density in clinically diagnosed or biomarker-confirmed AD cohorts.^14^^,^^15^ These discrepancies likely result from heterogeneity in diagnostic rigor, OCTA scan protocols, segmentation boundaries, systemic vascular comorbidities, and image-processing pipelines across studies.

Beyond methodological factors, disease stage may be an important determinant of whether OCTA-based density metrics capture AD-related changes. Studies reporting reduced vessel density have generally recruited participants with more advanced or longer-standing disease. Bulut et al.^28^ demonstrated significant correlations between retinal vascular density parameters and Mini-Mental State Examination (MMSE) scores, supporting the association between capillary rarefaction and worsening cognitive impairment. Our cohort, with mean disease durations of 3.7 ± 1.5 years for AD and 3.1 ± 1.3 years for MCI, may therefore represent a stage at which perfusion impairment is present, while capillary rarefaction remains below the detection threshold of conventional OCTA density metrics. The meta-analysis by Mathew et al.^15^ noted that retinal vascular alterations may occur even before significant cognitive decline, further suggesting that disease stage could contribute to variability in OCTA vessel density findings.

Moreover, vessel density metrics may be relatively insensitive indicators of early microvascular involvement, with retinal hemodynamic alterations potentially emerging prior to detectable changes in OCTA-derived capillary network density measures. The preserved RVD observed here, therefore, aligns with a growing body of evidence suggesting that static vascular metrics may not reliably capture early AD-related microvascular pathology. Consistent with this interpretation, the smaller effect sizes observed for structural vascular density metrics compared with hemodynamic parameters may partially explain the absence of significant group differences in these measures. This dissociation mirrors cerebral observations in AD, where reductions in cerebral blood flow often precede measurable atrophy or vascular loss.^8^^,^^29^ Retinal hemodynamic abnormalities may therefore represent an early hallmark of AD-related pathology, potentially offering a sensitive biomarker for early disease detection before overt neurodegeneration.^1^^,^^30^

While previous studies have emphasized structural retinal changes in AD, such as thinning of inner retinal layers or reduced OCTA vessel density metrics,^4^^,^^31^^,^^32^ the current results highlight the importance of evaluating blood flow directly.^10^^,^^33^ RBF, RCF, and RTP reflect dynamic aspects of perfusion and the microvasculature's ability to meet local metabolic demand.^6^^,^^7^ Their reduction, even in the context of preserved vessel density metrics, suggests that retinal hemodynamic abnormalities may occur independently of measurable alterations in OCTA-derived capillary network density.

Additional evidence of altered hemodynamic–morphometric relationships is provided by the absence of significant correlations between RBF and OCTA-derived vascular metrics (RVD, RVLD, CPD) in the AD + MCI group, even after adjusting for age. In contrast, cognitively normal controls exhibited meaningful structure–function associations, consistent with preserved coordination between retinal perfusion and vascular density metrics. This divergence supports the interpretation that the dissociation seen in AD and MCI reflects disease-specific disruption of neurovascular homeostasis, rather than age-related changes alone.^34^^,^^35^

This dissociation may arise from the convergence of pathologic processes implicated in AD, including microvascular remodeling, endothelial dysfunction, metabolic alterations, or breakdown of the blood–retinal barrier.^24^^,^^36^^,^^37^ These mechanisms could impair perfusion independently of capillary rarefaction, leading to functional deficits without corresponding changes in OCTA-derived capillary density metrics.

In support of this, CPD, which reflects the spatial complexity and fractal geometry of the perfused capillary network,^38^ was also unrelated to RBF or RTV across all groups. Unlike metrics such as vessel density or length density, CPD captures the topological organization of the vasculature.^38^^,^^39^ Its independence from perfusion-related parameters suggests that vascular topology and retinal blood flow may reflect partially distinct physiologic processes, particularly in early neurodegeneration.

Additionally, the lack of correlation between RBF, CPD, and RTV observed in both patient and control groups may reflect not only physiologic independence but temporal differences in the evolution of vascular and neuronal changes. While a study has reported reductions in both RBF and RTV in AD, these metrics may decline at different rates during disease progression.^7^ Retinal hemodynamic abnormalities may therefore precede measurable tissue atrophy or detectable alterations in OCTA-derived vascular density metrics.^40^ This temporal decoupling, along with the population variability, may help explain the inconsistent correlations observed in cross-sectional studies. Moreover, it remains unclear whether reduced perfusion is a cause or consequence of neurodegeneration, highlighting the need for longitudinal studies to elucidate these relationships.^41^

Notably, the current cohort primarily consisted of individuals in stages of disease that may precede the onset of detectable neurodegeneration. The observed retinal hemodynamic–morphometric profile, marked by reduced perfusion-related parameters despite preserved OCTA-derived vascular density metrics, may therefore represent an early biomarker pattern emerging prior to irreversible neuronal loss.

This study has several limitations. First, its cross-sectional design limits the ability to determine the temporal relationship between retinal microvascular dysfunction and neurodegenerative processes in AD. Second, although age differences between groups were addressed using age-adjusted analyses, residual confounding related to aging cannot be completely excluded. Third, BCVA was not systematically recorded for the cognitively normal control group, which limited direct comparison of visual acuity across all groups. However, all participants underwent slit-lamp examination to confirm clear optical media and met strict refractive and image-quality criteria to ensure reliable retinal imaging.

Fourth, although the present study incorporated quantitative hemodynamic and OCTA-derived morphometric metrics, all measurements were obtained under resting conditions and therefore cannot directly assess dynamic neurovascular regulatory responses such as flicker-evoked vasodilation or autoregulatory capacity. Future studies incorporating dynamic retinal imaging paradigms may allow more direct evaluation of neurovascular regulatory function in AD and MCI, complementing the hemodynamic and morphometric measures reported here. Finally, the study sample was relatively modest and recruited from a tertiary referral center, which may limit generalizability. Future longitudinal studies with larger cohorts will be important to confirm these findings and clarify the temporal relationship between retinal vascular dysfunction and neurodegeneration.

## Conclusions

This study provides a comprehensive multimodal characterization of retinal neurovascular alterations in individuals with AD and MCI, identifying a distinct retinal hemodynamic–morphometric profile marked by reduced RBF, RCF, and RTP, despite preserved OCTA-derived vascular density metrics and retinal tissue volume. These findings suggest that retinal perfusion abnormalities may emerge independently of, or prior to, measurable alterations in capillary network density metrics in early cognitive impairment.

The absence of significant associations between retinal blood flow and OCTA-derived vascular metrics in AD and MCI, in contrast to the relationships observed in cognitively normal controls, further suggests altered interactions between retinal hemodynamic and morphometric parameters in cognitive impairment. Together, these results highlight the value of multimodal retinal imaging for detecting early neurovascular abnormalities that may not be captured by OCTA-derived vascular density measures alone, supporting its potential role as a noninvasive biomarker framework for early disease detection and monitoring in AD and MCI.

## Supplementary Material

Supplement 1
