# Supplementary material for: Reduced Retinal Perfusion With Preserved Microvascular Network Density in Early Alzheimer's Disease: A Multimodal Imaging Study
Source: Transl Vis Sci Technol. 2026 Aug 11;15(8):7. doi: 10.1167/tvst.15.8.7 (PMC13480020; doi:10.1167/tvst.15.8.7)
Supplement: Supplementary file 1 [file tvst-15-8-7_s001.pdf]

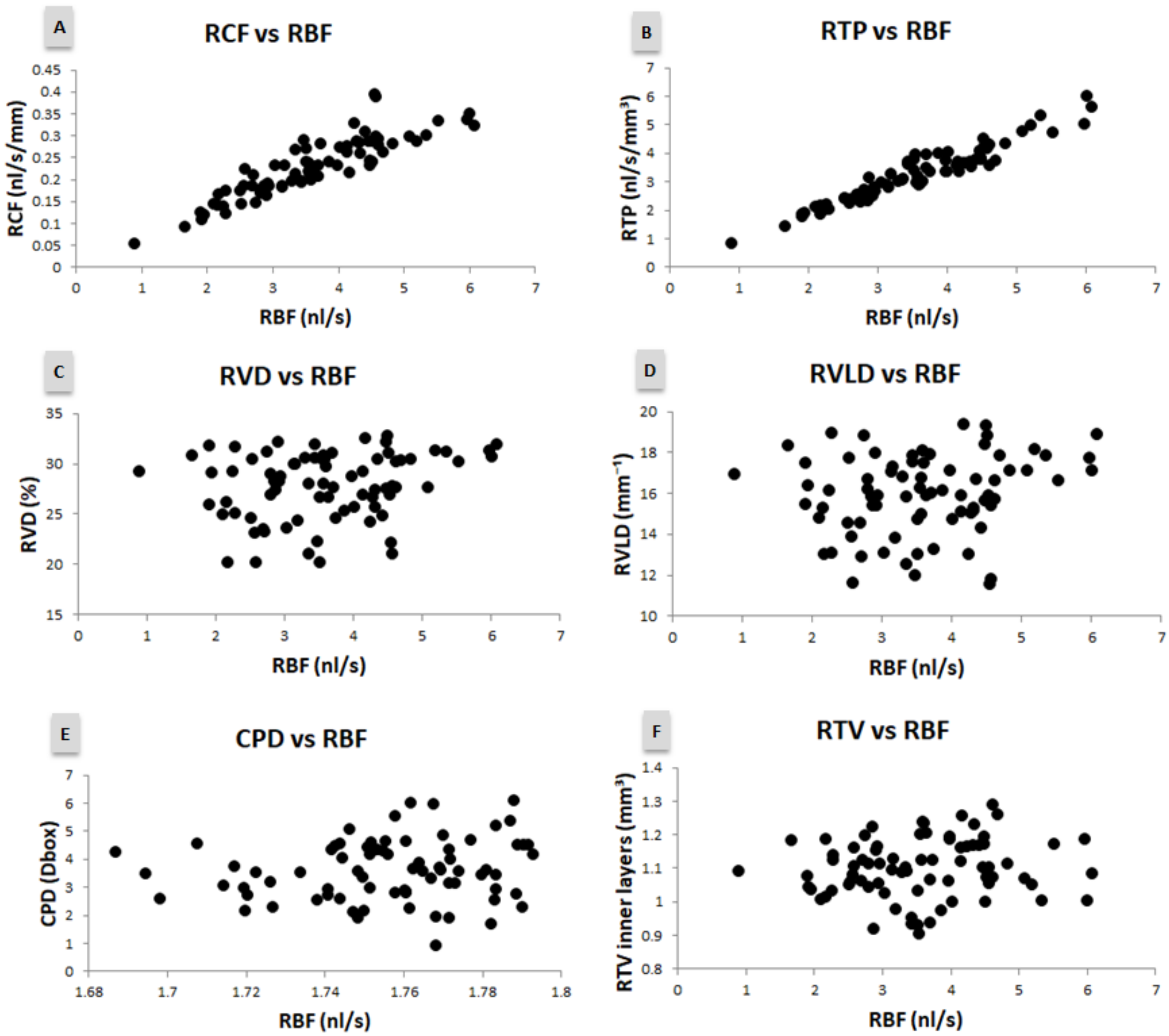

Supplementary Figure S1. Scatterplots illustrating pairwise relationships among primary retinal hemodynamic and structural variables across the study cohort (n = 82), provided to assess potential collinearity among the metrics.
